# Detecting coordinated regulation of multi-protein complexes using logic analysis of gene expression

**DOI:** 10.1186/1752-0509-3-115

**Published:** 2009-12-14

**Authors:** Einat Sprinzak, Shawn J Cokus, Todd O Yeates, David Eisenberg, Matteo Pellegrini

**Affiliations:** 1 UCLA-DOE Institute for Genomics and Proteomics, University of California Los Angeles, Los Angeles, CA, USA; 2 Department of Molecular, Cell, and Developmental Biology, University of California Los Angeles, Los Angeles, CA, USA; 3 Department of Chemistry and Biochemistry, University of California Los Angeles, Los Angeles, CA, USA; 4 Howard Hughes Medical Institute, University of California Los Angeles, Los Angeles, CA 90095, USA

## Abstract

**Background:**

Many of the functional units in cells are multi-protein complexes such as RNA polymerase, the ribosome, and the proteasome. For such units to work together, one might expect a high level of regulation to enable co-appearance or repression of sets of complexes at the required time. However, this type of coordinated regulation between whole complexes is difficult to detect by existing methods for analyzing mRNA co-expression. We propose a new methodology that is able to detect such higher order relationships.

**Results:**

We detect coordinated regulation of multiple protein complexes using *logic analysis *of gene expression data. Specifically, we identify gene triplets composed of genes whose expression profiles are found to be related by various types of logic functions. In order to focus on complexes, we associate the members of a gene triplet with the distinct protein complexes to which they belong. In this way, we identify complexes related by specific kinds of regulatory relationships. For example, we may find that the transcription of complex C is increased only if the transcription of both complex A AND complex B is repressed. We identify hundreds of examples of coordinated regulation among complexes under various stress conditions. Many of these examples involve the ribosome. Some of our examples have been previously identified in the literature, while others are novel. One notable example is the relationship between the transcription of the ribosome, RNA polymerase and mannosyltransferase II, which is involved in N-linked glycan processing in the Golgi.

**Conclusions:**

The analysis proposed here focuses on relationships among triplets of genes that are not evident when genes are examined in a pairwise fashion as in typical clustering methods. By grouping gene triplets, we are able to decipher coordinated regulation among sets of three complexes. Moreover, using all triplets that involve coordinated regulation with the ribosome, we derive a large network involving this essential cellular complex. In this network we find that all multi-protein complexes that belong to the same functional class are regulated in the same direction as a group (either induced or repressed).

## Background

In recent years, systematic experimental studies, such as those using TAP tag Mass-Spec techniques, have provided a draft map of yeast multi-protein complexes [1,2]. This map shows the composition of the quaternary protein structures in this model organism. The next challenge is to uncover which complexes work together to perform particular cellular tasks. One way to accomplish this is to detect the synchronized regulation of multi-protein complexes.

Coordinated regulation may be defined as a synchronous pattern of increased or reduced mRNA transcription of several cellular multi-protein complexes in response to a given perturbation. Such coordinated regulation of complexes is found when cellular function requires several complexes to be co-expressed or when other complexes need to be repressed for a given complex to function. For example, to achieve proper initiation of the translation process in eukaryotes, numerous cellular multi-protein complexes are regulated in a coordinated fashion. In this process, the initiation factor complexes eIF2, eIF3, and the cap-binding protein complex (eIF4f) associate to bind the ribosomal small subunit complex (40S) (reviewed in [3]). Another example involves the TOR complex 1 (Target Of Rapamycin), a conserved Ser/Thr kinase that regulates cell growth and metabolism in response to nutrients and stress. When nutrients are available, TOR activates complexes related to ribosome biogenesis, translation and nutrient import. In contrast, starvation inhibits TOR activity, thereby inducing various cellular responses such as cell arrest in the early G1 phase, inhibition of protein synthesis, nutrient transporter turnover, transcriptional changes, and autophagy. These responses are all mediated by multi-protein complexes [4,5].

Intricate relationships among genes and groups of genes (multi-protein complexes) are not captured by simple pairwise correlations; rather, higher order analysis is necessary to derive more detailed relationships. In the past few years diverse methods, such as binary and Bayesian networks, have been developed to derive gene networks (reviewed in [6]). However, these approaches aim to detect co-regulated expression modules among individual genes, while methods to detect co-regulation among groups of genes, such as multi-protein complexes, still need to be developed. In the present study, we apply *logic analysis *to gene expression data to identify gene triplets related by various types of logic functions [7]. Next, we combine these to study coordinated relationships among multi-protein complexes.

**Figure 1:**
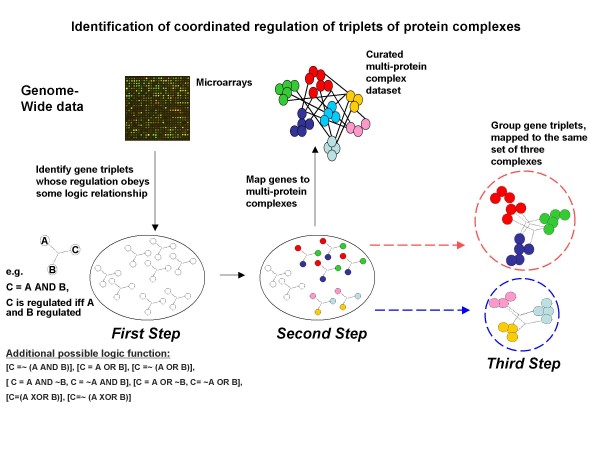
**Analysis outline**. We first apply logic analysis to microarray data to identify gene triplets whose regulation obeys one of the possible logic functions such as the AND function: C is regulated iff A and B are regulated (the functions are described in detail in Additional file 1: Table S1). Next, using a curated set of protein complexes [19,32], we map gene triplets onto complexes. Finally, we identify triplets of complexes and calculate their significance (see Methods section). The analysis enables us to predict coordinated regulation of these complexes.

**Figure 2:**
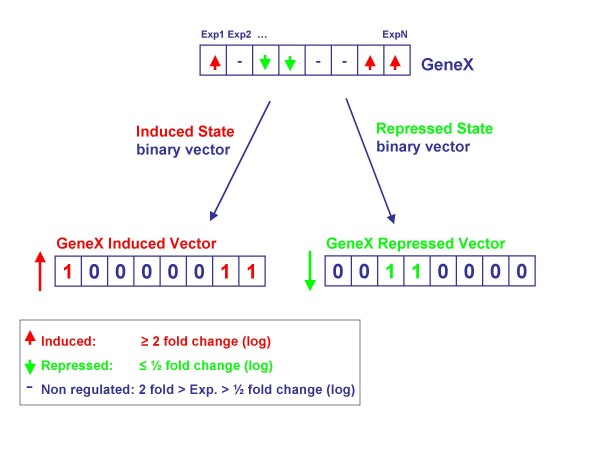
**Conversion of gene expression data into induced/repressed binary vectors**. Based on defined cut-offs, we determine whether each gene is induced or repressed or non-regulated in each condition. Next, we derived for each gene two vectors: induced and repressed state binary vectors. In the induced state binary vector, we assigned one to all experiments where the gene was induced, and otherwise zero. In the repressed state binary vector, we assigned one to all experiments where the gene was repressed, and otherwise zero.

Logic analysis is a method to relate triplets of genes/proteins by certain logic functions based on genomic data. All eight possible logic functions among triplets of genes can be found in Figure 1 and Additional file 1: Table S1. The triplet logic approach was introduced by Bowers *et al*. [7] and applied to genomic data in the form of phylogenetic profiles (described in [8]). Subsequently, logic analysis was also applied to find relations between the expression of two genes and disease state phenotypes [9]. In the current study, logic analysis is extended and modified for application to gene expression data. The original approach assigned a binary value (0 or 1) to a gene for each organism or gene expression experiment. In this work we use a three state model that describes genes as induced, repressed, or non-regulated. We construct two separate regulatory state vectors for each gene, where one vector describes whether a gene is induced or not over the set of experiments, while the other describes whether the gene is repressed or not (Figure 2). These vectors are then used to identify gene triplets whose regulation obeys logic functions [7]. For example: gene C is induced/repressed if and only if (iff) gene A is induced/repressed and gene B is induced/repressed. We also introduce a P-value for each gene triplet that quantifies the likelihood of obtaining this triplet by chance (see Methods). Next we grouped genes with the same logic function that mapped to the same set of three multi-protein complexes. This type of grouping enables us to infer how the coordinated regulation of these complexes occurs in the cell (Figure 1).

To explore the utility of our approach, we applied triplet logic analysis to yeast microarray data that measures the response of gene expression levels to environmental changes [10]. We identified genes whose regulation obeys triplet logic functions, and mapped these genes to distinct, multi-subunit complexes to infer coordinated regulation between the complexes. Among the many complexes inferred to have coordinated regulation, we discuss examples related to the biogenesis of the ribosome and support them with known regulatory data. In addition, we derive a cellular network of all complexes that have different triplet relationships with the ribosome. This network reveals that in stress conditions, all complexes belonging to the same functional classes are regulated in the same direction (induced/repressed). This observation may suggest the existence of global regulation of numerous cellular multi-protein complexes that belong to the same functional class.

## Results

### Identifying gene triplets whose regulatory patterns obey logic functions (Figure 1, first step)

**Figure 3:**
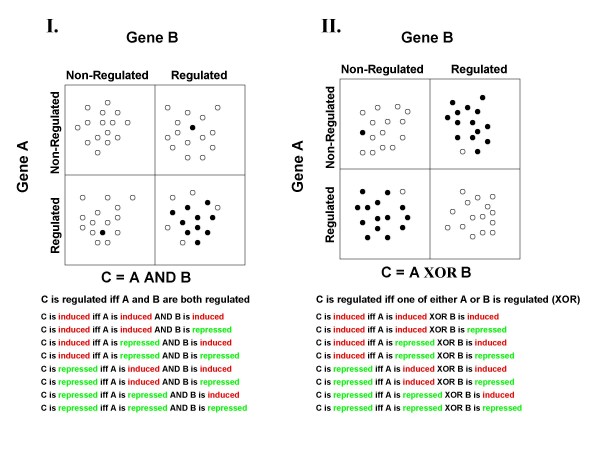
**Illustration of triplet logic relationships in which the regulation of a third gene is strongly correlated to some combined regulation of two other genes**. Each circle represents a single mRNA profile readout and its position indicates whether the expression of A and B are regulated. A filled circle implies that C is regulated, while an empty circle implies C is non-regulated. Tables **I**. and **II**. describe the two logic patterns that were analyzed in this study. Below each table is the logic statement and associated text. Each gene can be in three possible states: induced, repressed or unregulated. This expands the number of logical relationships we see. **I.) **The case of regulated expression described by the AND logic statement. We have eight possible different regulatory states for logic statement AND as shown in the figure. **II**.) The case of regulated expression described by the XOR logic statement. For this logic statement we also have eight possible regulatory states.

We applied logic analysis to expression data to identify gene triplets whose regulation obeys one of the eight possible logic functions (Additional file 1: Table S1 and Figure 3). The analysis was applied to data of Gasch *et al*. ([10]), which measure the expression of all *Saccharomyces cerevisiae *genes in response to various environmental stresses. Initially, we constructed two binary state vectors for each gene. One vector describes whether the gene is repressed and the other vector describes whether the gene is induced across of the microarray experiments; vectors were retained for analysis only if induction or repression was seen in at least 10% of experimental conditions (see Methods and Figure 2). This resulted in 2,969 (~ 25%) gene vectors, 45% of which represent the induced state while 55% represent the repressed state. Next, using these binary vectors, we analyzed all possible gene triplets. We identified about nine million potentially significant gene triplets, based on the associated uncertainty coefficient (U) and P-value. These thresholds were chosen to filter out triplets that are only related by pairwise correlations between two genes (see detailed description in Method section). Some of the gene triplets were significant under more than one type of logic function. In these cases we assigned to each gene triplet the most significant logic function as defined by the highest U value. This assignment reduced the number of non-redundant triplets for further grouping and analysis to 5,241,065.

The eight possible types of triplet logic relationships described earlier [7], occur with different frequencies (Additional file 1: Table S1). The four types (A AND !B, !A AND B), A XOR B, A OR B, and A AND B represented 53.5%, 30.6%, 15.2%, and 0.7% of the cases, while the remaining four types almost never occurred. We believe certain logic types are rare because the binary microarray data we are using is relatively sparse (it contains many more zeros than ones). As a result, only logic functions where f(0,0) = 0 are observed often, whereas functions where f(0,0) = 1 are not. Additional file 2: Figure S1 contains example heat maps of triplets of genes that obey the AND and XOR logic functions.

### Mapping gene triplets to multi-protein complexes (Figure 1 second step)

We mapped all gene triplets to complexes as described in Methods. We identified 40,521 triplets that were composed of genes that mapped to multi-protein complexes. Of these triplets, 412 (1%) mapped to a single multi-protein complex, 40,109 (99%) triplets mapped to at least two different complexes and about 90% mapped to three different complexes. As mentioned above, the U value was used to filter out gene triplets that are associated only by pairwise correlations (see Methods). That most gene triplets mapped to more than one complex supports our choice of this threshold.

### Grouping gene triplets that map to three complexes (Figure 1 third step)

Next we grouped together gene triplets obeying the same logic function and mapping to the same set of three complexes (Figure 1, third step). We restricted our analysis to two logic functions: XOR and AND. These two functions were abundant in our data and were judged to have more intuitive biological interpretations than the other logic types (Figure 3). The logic function AND yields 397 triplets of protein complexes. For each triplet of complexes we computed the significance of the finding based on the number of gene triplets that map to these complexes and computed a P value using the hypergeometric distribution (see Methods section). Out of these 397 triplets of protein complexes, 102 (25.7%) are significant (P ≤ 0.05 adjusted for Bonferroni correction). A total of 15,915 triplets of protein complexes were related through the logic function XOR, of which 729 (4.6%) are significant (P ≤ 0.05 adjusted for Bonferroni correction).

The significant triplets of protein complexes related through logic functions AND and XOR include 69 and 159 different protein complexes which are supported by 230 and 3,775 gene triplets respectively. The genes composing the triplets encode a subset of the subunits of each complex. This may be explained by the incompleteness of the microarry data (missing measurements in specific experiments) and the strict parameters we choose. To check if the subunits we identify are representative of the entire complex, we calculated the expression coherence between the subunits of a complex. We found that in all complexes that appear in our study, the expression was indeed coherent (Additional file 3: Table S2).

The list of all triplets of protein complexes which have coordinated regulation (under the AND and XOR logic functions) appears in Additional files 4 and 5: Tables S3 and S4. Below we discuss examples of the triplets of protein complexes whose synchronized regulation has been previously described in the literature, as well as novel predictions of co-regulation of complexes.

### Regulation of protein translation, autophagy degradation and N-linked glycosylation - examples of triplet complexes that have coordinated regulation obeying the AND logic function

**Figure 4:**
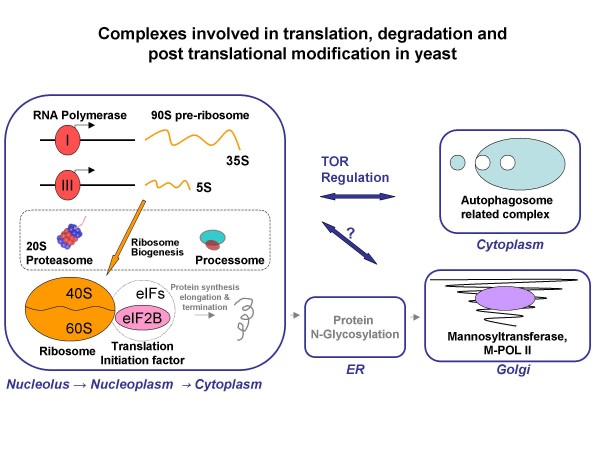
**Complexes involved in translation, degradation and post translational modification in yeast**. Schematic representation of different multi-protein complexes involved in processes related to translation, which were identified in several triplets of complexes predicted to be coordinately regulated. **RNA polymerase I and III **complexes are part of the transcriptional machinery that transcribe the ribosome precursors, 35S and 5S, that after proper processing form the **ribosome subunits (40S and 60S) **[35]. The **Processome **is involved in ribosome processing and maturation, as is the **20S Proteasome **[16]. The **eIF2B **(eukaryotic initiation factor 2B) complex is required for protein translation initiation and its regulation [36]. Protein translation initiation is the first step in protein synthesis and precedes the elongation and termination steps to complete polypeptide production. Another complex in this pathway is **Aut2/Aut7, authophagy related complex **which is active in the cytoplasm and responsible for degradation during environmental changes [5,37]. The **M-POL II, mannosyltransferase II **is the third complex enzyme in "mannan" modification of N-linked glycan processing in the Golgi apparatus. The two processes of translation and authophagy are both known to be mediated by the TOR complex [4,5].

Figure 4 is a schematic representation of various multi-protein complexes involved in processes related to translation. The figure caption provides a brief description of the function of the complexes whose co-regulation is described in the following sections.

### Ribosome large subunit - 60S, eIF2B initiation factor and RNA polymerase I/III

**Figure 5:**
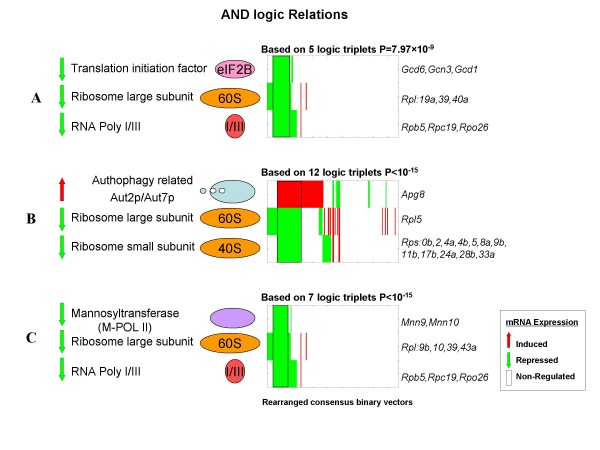
**Heat maps of examples of triplets of protein complexes predicted to have coordinated regulation obey the AND function**. Each heat map represents the mRNA expression level of the subunits of the three complexes, represented by consensus vectors (gene names appear on the right hand of the heat map). On top of each heat map is the number of gene triplets mapped to the same set of complexes and the significance of observing this by chance. The x-axis in the heat map indicates the different experiments involved in the analysis, which were optimally ordered for visualization of the logic relationship (these appear in the outlined rectangle). The subsets of stress conditions in which the transcription of all three complexes is coordinately regulated: (A) variable temperature shocks, amino-acid starvation, and stationary state long term, (B) heat shock, hydrogen peroxide, nitrogen depletion, diauxic shift (shift from anaerobic fermentation of glucose to aerobic respiration of ethanol), and stationary phase long term (in which the yeast cell's cell-cycle and growth are stopped), (C) heat shock, variable temperature shocks and stationary phase long term.

Our results reveal that the transcription of the 60S ribosomal large subunit decreases if and only if (IFF) the transcription of the eIF2B initiation factor **AND **RNA polymerase I/III are decreased as well. The three subunits of the RNA polymerase, RPB5, RPC19, and RPO26 that participate in this logic relation are components of both polymerase I and III. Figure 5(A) shows the subset of experiments (outlined rectangle) where the transcription of all three complexes decreases. Indeed, co-regulation between complexes involved in ribosome biogenesis (RNA polymerase I/III) and protein translation (eIF2B initiation factor) was shown recently to be mediated by TOR signaling, as reviewed in Wullschleger *et al*. [4]. In response to nutrients, TOR induces ribosome biogenesis, translation, and nutrient import, whereas stress conditions repress these functions [4]. Our results suggest the stress conditions tested in these experiments inhibit TOR signaling and this inhibition leads to the repression (either direct or indirect) of all three complexes. ChIP-chip data reveal that the genes encoding the subunits of Ribosome 60S, RNA polymerase and eIF2B are bound by overlapping sets transcription factors. Genes encoding RNA polymerase I/III subunits and ribosome large subunits are bound by the ABF1 transcription factor (ARS-Binding Factor 1), whereas genes encoding the RNA polymerase I subunit and the eIF2B subunit genes are bound by RPN4 (Regulatory Particle Non-ATPase) and DIG1 (Down-regulator of Invasive Growth).

### Ribosome 60S and 40S subunits and the autophagy related complex

We find that the transcription of the 60S ribosome large subunit decreases only when the transcription of the 40S subunit decreases **AND **the transcription of autophagy related complex increases. Figure 5(B), shows that in a subset of experiments the transcription of the autophagy-related dimer complex Aut2P/Aut7P is increased when the transcription of both of the ribosomal complexes, 40S and 60S is decreased. Although the relation includes only one subunit of the 60S ribosomal complex, it is known that ribosomal subunits are strongly co-expressed (average correlation coefficient of 0.87 (± 0.08) of 86% of possible pairs within the ribosome). All other subunits of the 60S ribosome were assigned lower scores due to incompleteness of the microarray data in the specified experiments and the strict parameters we choose. Aut2P/Aut7P has a role in protein degradation while the two ribosomal complexes 40S and 60S have a role in protein synthesis. That these two complexes have opposite function likely explains their opposite transcriptional regulation in this subset of experiments (outlined rectangle). In this example as in the previous one, the TOR signaling pathway is known to mediate both the translation and the autophagy processes. When the cell experiences stress conditions, the TORC1 complex is inhibited. This inhibition leads to decreased transcription of genes involved in translation and also leads to activation of the autophagy process [4,5].

We identified TFs that bind genes of both the 40S and 60S ribosomal complexes, but could not identify TFs that also bind genes encoding the Aut2/Aut7 complex, possibly because we employed strict filtering of the ChIP-chip data (see Methods). Moreover, we did find that genes encoding subunits of the TORC1 (TOR complex) and ribosomal 40S and 60S subunits are all bound by the REB1 (RNA polymerase I Enhancer Binding protein), PHO2 (PHOsphate metabolism regulator) and MSN4 (activated in stress conditions) TFs.

### Ribosome 60S, RNA polymerase I/III and Mannosyltransferase glycosylation complex

The transcription of the 60S ribosomal large subunit decreases only when the transcription of RNA polymerase I/III **AND **the M-POL II complex are both decreased. Figure 5(C) shows coordinated reduction in the transcription of the ribosome complex, the RNA polymerase complex (I/III), and the M-POL II complex in a subset of stress conditions (outlined rectangle). The M-POL II, mannosyltransferase II is the third complex enzyme in mannan modification of N-linked glycan processing (elongating the α (1,6) mannan backbone) in the Golgi apparatus. The importance of N-linked glycan processing is underscored by the fact that mannoproteins make up about 40% of the yeast cell wall [11-13]. The substrates of the POL-II enzyme are N-linked glycan modified proteins from the ER (Figure 4). Glycosylation in the ER has been shown to be important for many polypeptides to undergo proper or complete folding (reviewed in [14]). Thus, we expect tight regulation of ribosome translation in the cytoplasm, followed by modification of N-linked glycans (ER) and subsequent mannan modification of N-linked glycan by the M-POL II (golgi). The subset of stress conditions for which the transcription of these three complexes decreases (legend Figure 5) are all known to reduce overall protein synthesis.

We find that the ABF1 transcription factor binds to genes encoding subunits of all three complexes. Moreover, the YAP5 basic leucine zipper (bZIP) transcription factor was found to bind the genes encoding the ribosome 60S and the M-POL II subunits.

We are unaware of evidence in the literature of coordinated regulation between translation - related complexes and mannan modification in the golgi. Our analysis therefore generates a novel prediction supported by TF binding data and the known biological roles of the complexes.

### Ribosome synthesis and regulation - an example of coordinated regulation among complexes obeying the XOR logic function

One of the significant triplets of protein complexes that are related by an XOR (exclusive OR) logic function, involves the processome. The example presented here results from combining two triplets of protein complexes: processome, proteasome and the 60S and 40S ribosomal subunits. In this triplet, processome transcription decreases if the transcription of the ribosome (40+60S) decreases, **XOR **the transcription of the proteasome increases. Prior experimental studies of these three multi-protein complexes support the proposed logic relationship we find between these complexes. It has been suggested that the rRNA processome SSU (Small Subunit) complex has two roles in the maturation process of the pre-ribosome 90S [15]. The first role of the rRNA processome is carried out by its sub complex t-Utp (U3 proteins), which is recruited to the Pol I promoter upstream of the rDNA gene for transcription initiation. The second role of the processome is pre-rRNA cleavage of the pre-ribosome 90S before transcription is completed. In recent work with mammalian cells, Stavreva *et al*. found that complexes associated with pre rRNA processing factors are ubiquitinated and hence labeled for processing by the proteasome, a step essential for proper activity in ribosome maturation. One of the factors found to be ubiquitinated is fibrillarin, a yeast NOP1 homolog that is a subunit of the rRNA splicing processome [16]. As the processome was found to regulate its own activity [17], reduction of its abundance may lead to decrease of its own transcription. The co-regulation of these three complexes is reasonable given the proposed regulation mechanism by the proteasome.

**Figure 6:**
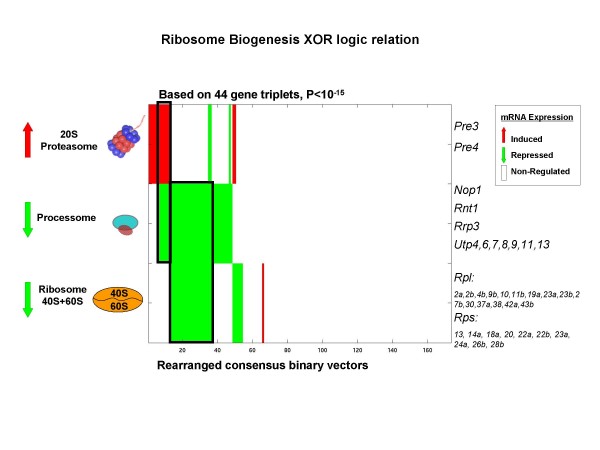
**Heat map of example of triplet of protein complexes predicted to have coordinated regulation obey the XOR function**. For a description of the heat map see the legend of figure 5. The subsets of stress conditions for which transcription of both the processome and ribosome decreases: heat-shock, dithiothrietol (DTT), AA starvation, nitrogen depletion and YPD long term stationary phase (the rightmost outlined rectangle). The subset of stress conditions for which proteasome transcription is induced while the processome transcription is reduced: response to 0.3 mM H_2_O_2 _in cells with deletions of stress induced TFs (the leftmost outlined rectangle).

Figure 6 presents the consensus mRNA expression vectors of the three complexes as a heat map showing the logic relationship between their transcription patterns. The subset of stress conditions for which the transcription of both the processome and ribosome decreases (describe at legend Figure 6) is likely to cause a drop in the "translation" rate. While the subset of stress conditions for which proteasome transcription is induced while the processome transcription is reduced might be related to processome degradation by the proteasome [16]. The relevant subsets of experiments in the second case, include response to 0.3 mM H_2_O_2 _in cells with deletions of stress induced TFs. In fact, it was shown that high H_2_O_2 _concentrations results an increase rate of ribosome biogenesis and maturation [18], substantiating our prediction. Two transcription factors were found to bind several of the genes encoding subunits of all three complexes: RAP1 (Repressor Activator Protein) and CBF1 (Centromere-Binding Factor).

### Cellular network of all complexes having different triplet relationships with the ribosome

**Figure 7:**
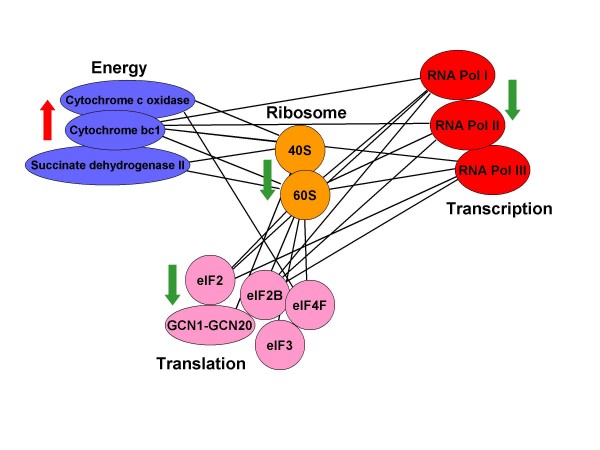
**Sub network of AND relations between the ribosome, energy, transcription and translation related complexes**. The sub-network was derived using all AND triplets that involve complexes associated with the ribosome (small and large subunits). Each group of complexes in the same functional class, as defined by MIPS functional categories [19], is colored differently. The arrow next to the functional classes represents the regulatory state of the subunits of the complexes: green - mRNA repressed, red - mRNA induced. Complexes in the "Transcription" category are the RNA polymerases I, II and III. Complexes in the "Translation" category are eIF2, eIF2B, eIF3, eIF4F translation initiation factors and GCN1-GCN20 regulate the translation elongation. Complexes in the "Energy" category are cytochrome bc1, cytochrome C oxidase and Succinate dehydrogenase II, all mitochondrial redox carriers.

By grouping together all predicted complex triplets that obey the same type of logic function (AND) and involve the ribosomal small or large subunits, we were able to generate a network. Figure 7 shows a subset of this network that includes complexes belonging to the "energy", "transcription" and "translation" functional classes (as defined by MIPS functional categories [19]). The figure shows that complexes belonging to the same functional classes are regulated in the same direction. In response to stress conditions, complexes belonging to the functional class "energy" are positively regulated (induced), while complexes belonging to the functional classes "transcription" or "translation" are negatively regulated (repressed). This result suggests that the regulation of different complexes may be determined by a master regulatory mechanism that differentially controls multi-protein complex expression, based on function.

## Discussion

In the current study we present a method that offers insights into how the regulation of numerous multi-protein complexes is coordinated. In this method, we apply logic analysis to microarray data to identify gene triplets whose transcription obeys logic functions. We then map these gene triplets to consistent sets of protein complexes. This approach allows us to infer statistically significant coordinated regulation among triplets of protein complexes. This mapping reduces the complexity associated with the analysis of gene triplets and increases the significance in our triplet identifications.

Typically, in the triplets of protein complexes we identify, only subsets of experiments are coordinately regulated among all complexes. Several approaches were previously used to identify genes which function together in subsets of experiments. Ihmels *et al*. developed the "signature algorithm", a clustering approach, to identify gene regulatory modules [20]. Segal *et al*. identified regulatory modules and their condition-specific regulators from gene expression data using a probabilistic method [21]. However, unlike these previous methods, the present work identifies higher order relationships between genes. In our work, we specifically focus on relationships between triplets of genes that are not evident when genes are examined in a pairwise fashion. To confirm this point, we analyzed pairwise correlations between the genes in our triplets of complexes and detected significant correlations (P ≤ 0.05) only between pairs of complexes but not among all three. This result was further confirmed by analyzing the rank of the three correlation coefficients among all possible pair-wise complex relations (see Additional file 6: Table S5).

The examples of triplets we discussed demonstrate the biological relevance of our findings. However it is difficult to find a suitable benchmark to globally validate our results since the complexes within our triplets may have distinct biological functions. One possible way to validate our approach is to use synthetic data. To this end, we generated synthetic triplets by creating two random 0/1 vectors and a third vector that matches one of the logical combinations of the pair. A thousand such synthetic triplets were generated to match one of the logic functions AND and XOR and uncertainty coefficients and P-values for each triplet were calculated using our program. As expected, all synthetic triplets were identified as significant (Additional file 7: Table S6). In order to study the robustness of our method, we measured the number of significant complex triplet relations that could be identified based on random subsets of the gene triplets. We found that for both logic types XOR and AND, the drop in significant complex triplets we identified is proportional to the size of the random fraction of logic gene triplet used (Additional file 8: Figure S2).

The microarray data we used in this study measures mRNA levels in yeast cells in response to environmental changes [10]. A recent study using affinity purification of endogenously formed ribosmes and the analysis of associated mRNAs with DNA microarray shows that in stress dependent conditions there is a coordination of transcriptome and translatome in yeast [22]. This recent finding indicates that the coordinated regulation we identified between triplets of complexes based on the mRNA levels of the encoding subunits, may also extend to their protein levels. By focusing on microarray data of environmental stresses we detected coordinated regulation among complexes centering on the ribosome. Because the ribosome is responsible for protein translation, a variety of mechanisms are required to regulate its biogenesis, especially under stress conditions. Similar results were reported in another study by Levy *et al*. which found that ribosome biogenesis genes responded more to changes in the environment and less to longer-term changes in growth rates [23]. Since the ribosomal subunits 40S and 60S (small and large subunits) are large complexes composed of 57 and 81 subunits respectively [19], the fact that we found many gene triplets that involve the ribosome is not surprising. Although these two subunits function together in the translation machinery, they are usually defined as two separate complexes that are not permanently associated throughout the entire translation process (reviewed in [24]). In addition, we find that these two subunits are independently regulated in different conditions (Figures 5 and 6).

Using all triplets that involve complexes associated with the ribosome (small and large subunits), we derived a network. We found that all multi-protein complexes that belong to the same functional class are regulated in the same direction (either induced or repressed) (Results and Figure 7). This result suggests that the regulation of different complexes may be determined by a master regulatory mechanism that differentially controls multi-protein complex expression, based on function.

In a study by Lichtenberg *et al*. the authors analyzed the dynamics of complex formation during the yeast cell cycle. The authors found that in many cases (mainly complexes related to replication transcription and cell cycle) only a few subunits of each complex are transcriptionally regulated in order to control the timing of the final assembly. The authors claimed that this general design principle of "just-in-time" assembly would have an advantage over "just-in-time" synthesis of entire complexes since only a few components need to be tightly regulated in order to control the timing of the final complex assembly [25]. In our study many of the complexes which we identified to have coordinated regulation with the ribosome are involved in transcription, translation and energy. When we measured the level of co-expression among the subunits of these complexes (Additional file 3: Table S2), we found that many of them exhibit highly coherent transcription (similar findings reported by Simonis *et al*. [26]). This may indicate that for complexes that are active across larger time scales, transcriptional regulation affects most subunits. This is different from the regulation observed in complexes involved in cell-cycle activity which need to function during specific time frames, and for which a "just-in-time" assembly mechanism is more suitable. In another study by Teichman *et al*. the authors found that for a few complexes (e.g. Ribosome, RNA Polymerase, Proteasome) subsets of subunits have conserved co-regulation between yeast and worm [27]. This evolutionary conservation may indicate that although those complexes exhibit highly coherent transcription regulation, tighter regulation might exist between a subset of all the subunits.

## Conclusions

The importance of studying relationships between different modules in a cell such as multi-protein complexes has been demonstrated in different studies [28-31]. In our approach we used pre-defined modules in the cell-- multi-protein complexes-- [1,2] and identified triplets of these whose regulation obeys logic functions. This approach allows us to uncover coordinated regulation among complexes. Understanding this regulation allows us to infer higher-level modes of cellular function and also provides insight into the biological mechanisms underlying coordination between complexes. Our logic analysis can be applied to any transcriptional profiling data. Furthermore, this same methodology may be applied to other types of pre-defined functional modules, such as metabolic pathways.

## Methods

### Identification of gene triplets whose regulation obeys one of the eight possible logic functions

Gene triplets were identified using the entropy measure described in Bowers *et al*. ([7,9]). The uncertainty coefficient (U) we have used is a measure that can relate two profiles X and Y.

Where H(X) and H(Y) are the Shannon entropies for vectors X and Y respectively, n is the number of states in our data (in binary data we have two states 0 and 1), and P(i) is the frequency of each state i in our data. H(X, Y) is Shannon's entropy for the joint distribution between genes X and Y, where m is the number of states of the joint distribution between A and B (in binary data we have four states: 00, 01, 10, 11), and P(j) is the frequency of each state j in our data.

The uncertainty coefficient U is bounded between 0 and 1. When U = 0, X and Y are not related (independent) and when U = 1 it means that X is fully related to Y. More intuitively we can say that U is the fraction of information about X which can be learned if we know Y.

For all possible triplets of genes, we calculated the value U as the degree to which some logic combination of two vectors, A & B, describes a third vector C.

We also computed P-values which estimate the significance of the uncertainty coefficients (U) compared to those of random triplets. Our random model consisted of shuffled vectors with pair wise distributions maintained. Given three genes A, B and C whose regulation obeys a logic function with a specific *U(C|f(A,B)) *score, all three vectors A, B and C were randomized while keeping pairwise vector distributions of CA and CB constant.

In this study, we wish to identify instances where a logic combination of two vectors, A and B, have a significant *U(C|f(A,B))*, and P-value ≤ 10^-5 ^to describe vector C, while the individual vectors A and B alone have lower scores.

We require that the pair-wise uncertainty coefficients *U(C|A) *and *U(C|B) *be smaller by an amount X than the triplet uncertainty coefficient *U(C|f(a,b))*. This criterion was chosen in order to identify gene triplets which are not related by pair-wise correlations, as in the approach of Bowers *et al*. [7]. In this study we chose X = 0.1. Using a Bonferroni correction, P-value ≤ 10^-5 ^corresponds to a false discovery rate of 7.3%.

An extra filtering step was added to find more meaningful gene triplets whose co-expression was identified in a minimum of 10 experiments (5%) which match the logic combination of A and B, *f(A,B) *and vector C. We also required that this minimum number of experiments be higher than the number of experiments where only gene C or *f(A,B) *are regulated, to increase our confidence in the ternary over the pairwise relationships.

As some of the gene triplets matched to more than one type of logic function, we retained for each gene triplet only the logic function with the higher *U(C|f(A,B))*.

More details and exact mathematical formulation can be found in the Additional file 9: Supplemental methods.

The code for identifying logic triplets was implemented in C++ running on OS X and is available upon request.

### Mapping gene triplets to the same set of three complexes

In order to identify coordinated regulation among complexes using gene triplets, we first mapped genes onto complexes. For this mapping we used the MIPS curated complex database [19] and added non-redundant curated complexes (unpublished data) from the IntAct group [32]. We removed ambiguous complexes from this data, leaving us with 324 multi-proteins complexes involving 1,462 genes. In the next step we identified multiple gene triplets that mapped to the same set of three complexes (Figure 1, second and third step). The data from MIPS is based on the concept of complexes as static entities, and has a low number of subunits which are shared by more then one complex.

#### Identifying significant triplets of protein complexes predicted to have coordinated regulation

In order to check how significant the complex triplets are, we calculated the probability of obtaining the same number of gene triplets by chance using the hyper-geometric distribution:

Where *x *is the number of gene triplets that map to the same set of three complexes, *N *is the total number of gene triplets whose regulation obeys a logic function, *k *is the number of all theoretically possible gene triplets mapped to the same set of three complexes and *M *is all possible (theoretical) gene triplets mapped to any one of all possible sets of three complexes. We then computed the cumulative probability of observing x or more triplets.

### Yeast ChIP-chip data

In order to identify genes that bind the same transcription factors, we used ChIP-chip data [33]. The data contain measurements of transcription factor (TFs) target genes which were identified by binding assays. In order to extract out the more reliable TF binding data, we have used MacIsaac *et al*. [34] filtered data which identifies genes with conserved sequence elements among three *Saccharomyces *species. The subset we have used (P < = 0.005) includes 116 transcription factors and their 5,752 gene targets.

## Abbreviations

TF: transcription factor; IFF: if and only if; ChIP-chip: Chromatin ImmunoPrecipitation-chip; TOR: Target Of Rapamycin;

## Authors' contributions

ES conceived the study, preformed the computational analysis, analyzed data and wrote the manuscript. SJC conceived and implemented the statistical models and wrote the supplementary methods sections. TOY helped to design the study revise the manuscript. DE and MP jointly conceived the study, advised ES and revised the manuscript. All authors read and approved the final manuscript.

## Supplementary Material

Additional file 1 Table S1: Eight possible logic functions and their frequencies in our data. Click here for file

Additional file 2 Figure S1: Heat map examples of triplets of genes that obey logic functions AND (A) and XOR (B) Click here for file

Additional file 3 Table S2: Expression coherence of complexes found to have coordinated regulation with the Ribosome. Click here for file

Additional file 4 Table S3: Identified triplet complexes that have coordinated regulation obeying the AND logic function Click here for file

Additional file 5 Table S4: Identified triplet complexes that have coordinated regulation obeying the XOR logic function Click here for file

Additional file 6 Table S5: Pairwise correlations between complexes found to have coordinated regulation in examples appear in results section. Click here for file

Additional file 7 Table S6: Method validation using synthetic gene triplets that obey logic functions. Click here for file

Additional file 8 Figure S2: Significant logic complex relations identified while using subset of gene triplets. Click here for file

Additional file 9 Supplemental methods: Detailed overview of determination of selected logic triplets. Mathematical development of efficient direct computation of needed p-values. Computational considerations for efficient, accurate calculation of p-values. Click here for file
