# Supplementary material for: Detecting coordinated regulation of multi-protein complexes using logic analysis of gene expression
Source: BMC Syst Biol. 2009 Dec 14;3:115. doi: 10.1186/1752-0509-3-115 (PMC2804736; doi:10.1186/1752-0509-3-115)
Supplement: Additional file 1 — Table S1: Eight possible logic functions and their frequencies in our data. [file 1752-0509-3-115-S1.DOC]

**Table S1**: Logic functions and frequencies in our data.

| **Type** | **Logic Function** | **Logic statement** | **Gene triplets obey logic function (percentage)*** |
| --- | --- | --- | --- |
| AND | C = A **AND** B | C is regulated if and only if (iff) A and B are both regulated | 0.7% |
| !AND | C =~ (A **AND** B) | C is regulated iff A non-regulated and B non-regulated | 0% |
| OR | C = A **OR** B | C is regulated iff A is regulated or B is regulated | 15.2% |
| !OR | C =~ (A **OR** B) | C is regulated iff A is non-regulated or B is non-regulated | 0% |
| A AND !B, !A AND B | C = A **AND** ~B,  C = ~A **AND** B | C is regulated iff A is regulated and B non-regulated  C is regulated iff A is non-regulated and B regulated | 53.5% |
| A OR !B, !A OR B | C = A **OR** ~B,  C= ~A **OR** B | C is regulated iff A is regulated or B non-regulated  C is regulated iff A is non-regulated or B regulated | ~0% |
| XOR | C=(A **XOR** B) | C is regulated iff one of either A or B is regulated | 30.6% |
| !XOR | C=~ (A **XOR** B) | C is regulated iff A and B are both regulated or A and B are both non-regulated | ~0% |

*Percentage of gene triplets obey logic type out of total 5,241,065.
