# Supplementary material for: Detecting coordinated regulation of multi-protein complexes using logic analysis of gene expression
Source: BMC Syst Biol. 2009 Dec 14;3:115. doi: 10.1186/1752-0509-3-115 (PMC2804736; doi:10.1186/1752-0509-3-115)
Supplement: Additional file 2 — Figure S1: Heat map examples of triplets of genes that obey logic functions AND (A) and XOR (B) [file 1752-0509-3-115-S2.PPT]

## Slide 1
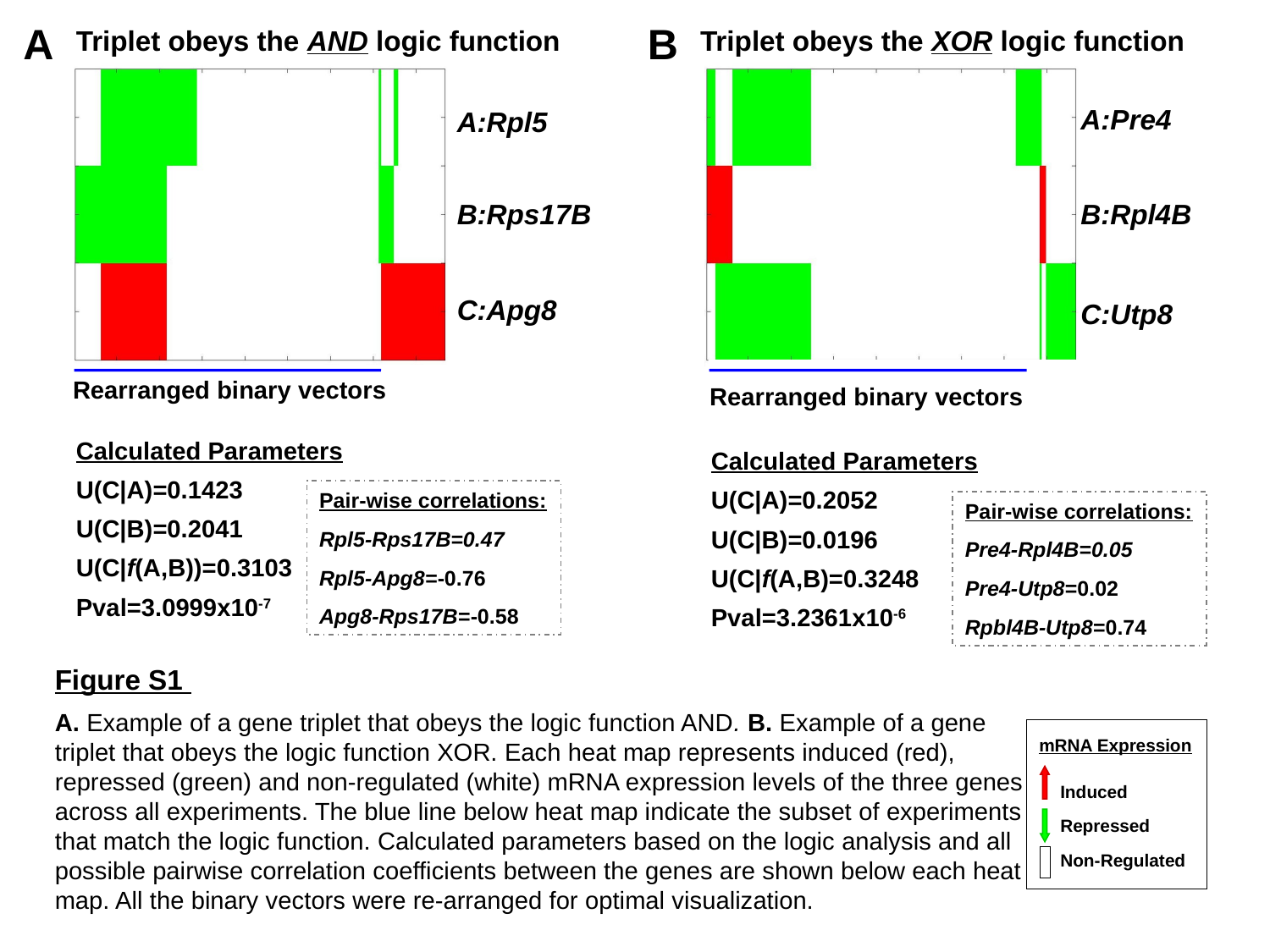

A
B
Triplet obeys the AND logic function
Triplet obeys the XOR logic function
Rearranged binary vectors
A:Pre4
A:Rpl5
B:Rpl4B
B:Rps17B
C:Apg8
C:Utp8
Rearranged binary vectors
Calculated Parameters
U(C|A)=0.1423
U(C|B)=0.2041
U(C|f(A,B))=0.3103
Pval=3.0999x10^-7^
Calculated Parameters
U(C|A)=0.2052
U(C|B)=0.0196
U(C|f(A,B)=0.3248
Pval=3.2361x10^-6^
Pair-wise correlations:
Rpl5-Rps17B=0.47
Rpl5-Apg8=-0.76
Apg8-Rps17B=-0.58
Pair-wise correlations:
Pre4-Rpl4B=0.05
Pre4-Utp8=0.02
Rpbl4B-Utp8=0.74
Figure S1
A. Example of a gene triplet that obeys the logic function AND. B. Example of a gene triplet that obeys the logic function XOR. Each heat map represents induced (red), repressed (green) and non-regulated (white) mRNA expression levels of the three genes across all experiments. The blue line below heat map indicate the subset of experiments that match the logic function. Calculated parameters based on the logic analysis and all possible pairwise correlation coefficients between the genes are shown below each heat map. All the binary vectors were re-arranged for optimal visualization.
mRNA Expression
Induced
Repressed
Non-Regulated
