# Supplementary material for: Detecting coordinated regulation of multi-protein complexes using logic analysis of gene expression
Source: BMC Syst Biol. 2009 Dec 14;3:115. doi: 10.1186/1752-0509-3-115 (PMC2804736; doi:10.1186/1752-0509-3-115)
Supplement: Additional file 3 — Table S2: Expression coherence of complexes found to have coordinated regulation with the Ribosome. [file 1752-0509-3-115-S3.DOC]

**Table S2:** Expression coherence of complexes which were found to have coordinated regulation with the Ribosome.

| Complex | Number Subunits | Percent pairs with correlation coefficient* above 0.5^a^ | Mean correlation coefficient (±STD)^b^ |
| --- | --- | --- | --- |
| Ribosome large subunit 60S | 81 | 86% | 0.87±0.083 |
| Ribosome small subunit 40S | 57 | 93% | 0.85±0.102 |
| RNA Pol I | 14 | 82% | 0.78±0.107 |
| RNA Pol II | 13 | 44% | 0.71±0.116 |
| RNA Pol III | 17 | 88% | 0.72±0.104 |
| eIF2 | 3 | 100% | 0.88±0.023 |
| eIF2B | 5 | 100% | 0.82±0.049 |
| eIF3 | 7 | 71% | 0.81±0.042 |
| eIF4F | 3 | 33% | 0.71±0.001 |
| GCN1-GCN20 | 2 | 100% | 0.81±0.000 |
| Cytochrom bc1 | 10 | 80% | 0.76±0.105 |
| Cytochrom c oxidase | 12 | 42% | 0.76±0.113 |
| Succinate dehydrogenase II | 4 | 100% | 0.86±0.065 |

^*^Pearson correlation coefficient calculated for all possible pairs using the Gasch *et al.* dataset

^a^ Percent pairs from all possible pairs within complex which their expression level have correlation coefficient above 0.5.

^b^ Mean and standard-deviation (STD) of all pairs within complex, which their expression level correlation coefficient above 0.5
