# Supplementary material for: Detecting coordinated regulation of multi-protein complexes using logic analysis of gene expression
Source: BMC Syst Biol. 2009 Dec 14;3:115. doi: 10.1186/1752-0509-3-115 (PMC2804736; doi:10.1186/1752-0509-3-115)
Supplement: Additional file 6 — Table S5: Pairwise correlations between complexes found to have coordinated regulation in examples appear in results section. [file 1752-0509-3-115-S6.DOC]

**Table S5**

| **triplet complexes that have coordinated regulation obeying logic function** | **All possible complex pairs** | **Mean Correlation Coefficient- X^a^** | **Percentile – Significance^b^** | **Triplet complex rank^c^** |
| --- | --- | --- | --- | --- |
| **AND:** **Ribosome large subunit, RNA polymerase I/III , eI2FB initiation factor** |  |  |  | 4.3825e+04 |
|  | eIF2B - RiboLarge | 0.768 | 0.079 |  |
|  | eIF2B – RNAPol13 | 0.815 | 0.047* |  |
|  | RiboLarge – RNAPol13 | 0.820 | 0.044* |  |
| **AND: Autophagy related complex, Ribosome large, small subunits** |  |  |  | 4.2995e+04 |
|  | Atuophagy - RiboLarge | -0.765 | 0.01* |  |
|  | Atuophagy - RiboSmall | -0.667 | 0.058 |  |
|  | RiboLarge - RiboSmall | 0.558 | 0.275 |  |
| **AND: Ribosome large subunit, RNA polymerase I/III, Mannosyltransferase glycosylation complex - M-POL II** |  |  |  | 1.1277e+05 |
|  | RiboLarge - RNAPol13 | 0.778 | 0.071 |  |
|  | RiboLarge - MPolII | 0.749 | 0.095 |  |
|  | RNAPol13 - MPolII | 0.765 | 0.081 |  |
| **Processome IFF Ribsome XOR 20 S Proteasome** |  |  |  | 1.6144e+06 |
|  | Processome - Proteasome | -0.076 | 0.388 |  |
|  | Processome - RiboLS | 0.718 | 0.122 |  |
|  | Proteasome - RiboLS | 0.104 | 0.528 |  |

**^a^** The mean of pairwise correlation coefficient values between the genes in our triplets (we included only genes having two fold change in expression level in at least 15 experiments as we used in the logical analysis)

**^b^** The percentile is calculated as [Number of gene pairs with higher (Positive) or lower (Negative) correlation coefficient than X out of Total^c^]/Total^c^ **, *** indicate significant value (<=0.05)

**^c^** The triplet rank is calculated using the gamma function which is calculating the P*, probability to get value less then P1*P2*P3 (The three pair wise percentile) multiple by all possible triplet of complexes in our set (224^3/2)
