# Supplementary material for: Detecting coordinated regulation of multi-protein complexes using logic analysis of gene expression
Source: BMC Syst Biol. 2009 Dec 14;3:115. doi: 10.1186/1752-0509-3-115 (PMC2804736; doi:10.1186/1752-0509-3-115)
Supplement: Additional file 7 — Table S6: Method validation using synthetic gene triplets that obey logic functions. [file 1752-0509-3-115-S7.DOC]

**Table S6**

**Method validation using synthetic gene triplets that obey logic functions**

|  | **AND*** | **XOR*** |
| --- | --- | --- |
| ***U(c|a)*** | 0.38245 (±0.041) | 0.00423 (±0.006) |
| ***U(c|b)*** | 0.38563 (±0.040) | 0.00454(±0.006) |
| ***U(c|f(a,b)*** | 1.0 | 1.0 |
| ***P-value*** | 2.437×10^-8^ (±1.725×10^-7^) | 1.144×10^-49^ (±8.319×10^-49^) |

*1,000 synthetic triplets were created by generating two random 0/1 vectors (of size 173) and the third vector that matches the logic function of the two random vectors. If the same triplet can be explained by more than one logic function, we assigned it the logic function with the highest *U(c|f(a,b)* (see Methods section). The values in the table are the average calculated parameters and calculated P-value for all synthetic triplets.
