# Supplementary material for: Detecting coordinated regulation of multi-protein complexes using logic analysis of gene expression
Source: BMC Syst Biol. 2009 Dec 14;3:115. doi: 10.1186/1752-0509-3-115 (PMC2804736; doi:10.1186/1752-0509-3-115)
Supplement: Additional file 8 — Figure S2: Significant logic complex relations identified while using subset of gene triplets. [file 1752-0509-3-115-S8.DOC]

**Figure S2:**

**Fraction of significant logic complex relations identified**

**using subsets of gene triplets**

The error bar for each point is computed from three random replicates of each subset size.
