# Supplementary material for: Detecting coordinated regulation of multi-protein complexes using logic analysis of gene expression
Source: BMC Syst Biol. 2009 Dec 14;3:115. doi: 10.1186/1752-0509-3-115 (PMC2804736; doi:10.1186/1752-0509-3-115)
Supplement: Additional file 9 — Supplemental methods: Detailed overview of determination of selected logic triplets. Mathematical development of efficient direct computation of needed p-values. Computational considerations for efficient, accurate calculation of p-values. [file 1752-0509-3-115-S9.PDF]

§1. Detailed overview of determination of selected logic triplets

Let integer $n \geq 1$ and vector $X = (X_1, \ldots, X_n)$ with n entries (*length* n) be given. The *range of* X is the set $r(X) := \{ X_i \mid i \in 1..n \}$ of distinct elements of X, the *histogram of* X is the function $h_X \colon r(X) \to 1..n$ with $h_X(x) :=$ the number of times x appears in X, and the *pdf of* X is the function $p_X \colon r(X) \to (0, 1]$ with $p_X(x) :=$ the fraction of times x appears in X (i.e., $h_X(x)/n$). The *entropy of* X *in bits* is

$$H(X) := -\sum_{x \in r(X)} p_X(x) \cdot \lg(p_X(x)) \geq 0$$

where lg is base 2 logarithm. $H(X)$ is a function of h_X, it is zero exactly when all elements of X are the same, and it increases as h_X becomes "more uniform."

Given a second vector Y of the same length as X and assuming for the moment that not all elements of X are the same, the *"uncertainty coefficient" of* X *given* Y is

$$U(X \mid Y) := \frac{H(X) + H(Y) - H(XY)}{H(X)} \in [0, 1]$$

where XY is the vector of length n with $(XY)_i = (X_i, Y_i) \in r(X) \times r(Y)$ for $i \in 1..n$. Note that h_X and h_Y are determined given h_{XY}. Roughly, $U(X \mid Y)$ is the fraction of X's randomness Y determines. $U(X \mid Y) = 0$ when corresponding entries of X and Y are independent in the sense of probability, while $U(X \mid Y) = 1$ when there is a deterministic function that gives X when applied elementwise to Y. (The property "is a deterministic function of" is not symmetric in general; $U(X \mid Y)$ and $U(Y \mid X)$ are generally different. Also, "certainty coefficient" might be a better name for U as it increases as X is *more* — rather than less — determined given Y, but the name is traditional.) In this work, $U(X \mid Y) = 1$ when $H(X) = 0$ (in this case, elements of X are necessarily simultaneously both independent and deterministic functions of the elements of Y and there is no natural value for $U(X \mid Y)$ to take). Note that $U(X \mid Y)$ is a function of h_{XY}.

In this work, at any moment one generally has three observed experimental binary $\{0, 1\}$–vectors A, B, and C (of the same length $n \geq 1$) and one of ten Boolean functions (see below) $f \colon \{0, 1\} \times \{0, 1\} \to \{0, 1\}$. Writing $f(A, B)$ for the "predicting" binary vector of length n with $(f(A, B))_i = f(A_i, B_i)$, a useful measure of how well A and B explain C according to relationship f is $U(C \mid f(A, B))$.

To judge a specific value of $U(C \mid f(A, B))$, it is helpful to compare it to the distribution of $U(C' \mid f(A', B'))$ values when A', B', C' are random binary vectors of length n with the same lower–order relationships of concern as A, B, C. Specifically, the observed U is compared to U from all uniform random triplets of binary vectors (A', B', C') after conditioning on $h_{A'} = h_A$, $h_{B'} = h_B$, $h_{C'} = h_C$, $h_{A'C'} = h_{AC}$, and $h_{B'C'} = h_{BC}$ (after experimentation, $h_{A'B'}$ was not directly constrained) or, equivalently, simply $h_{A'C'} = h_{AC}$ and $h_{B'C'} = h_{BC}$. Thus, one has p–value

$$p_f(A, B, C) := \Pr(U(C' \mid f(A', B')) \geq U(C \mid f(A, B)) \text{ when } A', B', C' \text{ are as above}) \in (0, 1],$$

which becomes smaller as A and B better explain C by relationship f compared to chance. Accurate but efficient computational calculation of such p–values in the present application requires mathematical manipulation and careful computational implementation as detailed below. In the main text, only relationships with $p_f \leq 10^{-5} =: \rho$ were considered.

There are ten Boolean functions $f(x,y)$ of genuinely two (and not fewer) variables.

$f(0,0)$	$f(0,1)$	$f(1,0)$	$f(1,1)$	$f(x,y) =$	"Type"	$\overline{f(x,y)}$
0	0	0	1	$\text{AND}(x,y) = x \wedge y =: f_1\ (x,y)$	type 1	type 2
1	1	1	0	$\text{OR}(\overline{x},\overline{y}) = \overline{x} \vee \overline{y} =: f_2\ (x,y)$	type 2	type 1
0	1	1	1	$\text{OR}(x,y) = x \vee y =: f_3\ (x,y)$	type 3	type 4
1	0	0	0	$\text{AND}(\overline{x},\overline{y}) = \overline{x} \wedge \overline{y} =: f_4\ (x,y)$	type 4	type 3
0	0	1	0	$\text{AND}(x,\overline{y}) = \overline{y} \wedge x =: f_{5'}\ (x,y)$	type $5'$	type $6''$
0	1	0	0	$\text{AND}(\overline{x},y) = \overline{x} \wedge y =: f_{5''}(x,y)$	type $5''$	type $6'$
1	0	1	1	$\text{OR}(x,\overline{y}) = \overline{y} \vee x =: f_{6'}\ (x,y)$	type $6'$	type $5''$
1	1	0	1	$\text{OR}(\overline{x},y) = \overline{x} \vee y =: f_{6''}(x,y)$	type $6''$	type $5'$
0	1	1	0	$\text{XOR}(x,y) = x \oplus y =: f_7\ (x,y)$	type 7	type 8
1	0	0	1	$\text{EQV}(x,y) = (x \equiv y) =: f_8\ (x,y)$	type 8	type 7

Types 1, 2, 3, 4, 7, and 8 are symmetric in their arguments; types $5'$, $5''$, $6'$, and $6''$ are not. However, $f_{5''}(x,y) = f_{5'}(y,x)$ and $f_{6''}(x,y) = f_{6'}(y,x)$, so that to find all relationships of interest among g profiles numbered $0..(g-1)$, one may (as is done here) enumerate (a,b,c,f) with f one of the ten Boolean functions above and distinct integers a, b, $c \in 0..(g-1)$ such that $a < b$, keeping just those that pass the p–value threshold ρ and rewriting types $5''$ as $5'$ (referring to both as "type 5") and $6''$ as $6'$ ("type 6"). As $U(X \mid Y) = U(X \mid \overline{Y})$ for all Boolean vectors X and Y of the same positive length, either both or neither of (a,b,c,f) and $(a,b,c,\overline{f})$ will pass ρ. If both pass, the one with fewer disagreements bitwise between C and the predicting vector is retained; if there is a tie (which cannot happen if n is odd, as with $n = 173$ of the main text), both are kept.

Additional filters are applied:

(1) $U(C \mid f(A,B)) \geq U(C \mid A) + \gamma$,
(2) $U(C \mid f(A,B)) \geq U(C \mid B) + \gamma$,
(3) $s := \#\{\, i \in 1..n \mid C_i = 1 \text{ and } f(A_i,B_i) = 1 \,\} \geq \tau$,
(4) $\#\{\, i \in 1..n \mid C_i = 0 \text{ and } f(A_i,B_i) = 1 \,\} < s$, and
(5) $\#\{\, i \in 1..n \mid C_i = 1 \text{ and } f(A_i,B_i) = 0 \,\} < s$.

The combination of (1) and (2) requires that C be better predicted from A and B together rather than just A alone or B alone (as the goal is to discover trinary and not pairwise relationships); γ was taken to be 0.1. Due to the sparsity of 1's in the profiles of the discretized version of the dataset of the main text, (3) was imposed to focus on relationships with the number $s \in 0..n$ of correct predictions on experimental positives (rather than the far more numerous and less interesting experimental negatives) being substantial; τ was taken to be 10. To further increase confidence as to the existence of a relationship, the combination of (4) and (5) requires s to separately exceed any disagreements between C and the predicting vector in each of the two ways they can disagree.

Finally, collect surviving logic triplets (a,b,c,f) into groups, each group consisting of those that share $\{a,b,c\}$ (as an unordered set of profile indices). In each group, retain only the logic triplet of highest $U(C \mid f(A,B))$; ties were broken arbitrarily.

§2. Mathematical development of efficient direct computation of needed p–values

Fix integer $n \geq 1$ and put $\Xi := \{0,1\}^n \times \{0,1\}^n \times \{0,1\}^n$, the set of all triplets of length n binary vectors. Given a Boolean function $f\colon \{0,1\} \times \{0,1\} \to \{0,1\}$ and $(A,B,C) \in \Xi$, to compute desired p–value

$$p_f(A,B,C) = \frac{\#\left\{ (A',B',C') \in \Xi \;\middle|\; \begin{array}{c} h_{A'C'} = h_{AC},\ h_{B'C'} = h_{BC}, \text{ and} \\ U(C' \mid f(A',B')) \geq U(C \mid f(A,B)) \end{array} \right\}}{\#\{\, (A',B',C') \in \Xi \mid h_{A'C'} = h_{AC} \text{ and } h_{B'C'} = h_{BC} \,\}} \qquad (\dagger)$$

by direct enumeration of members of Ξ is typically computationally infeasible, as $\#\Xi = 2^{3n} = 8^n$ and this (for example) exceeds 10^{156} when $n = 173$. Further, such would be for calculation of a single p–value, and to discover all relationships for, e.g., 3,000 profiles requires computation of some $3{,}000 \cdot 2{,}999 \cdot 2{,}998/2 \cdot 10/2$ — more than 67 billion — p–values.

However, because $h_{A'C'}$, $h_{B'C'}$, and $U(C' \mid f(A', B'))$ are all easily computed (see below) functions of $h_{A'B'C'}$ (where $A'B'C'$ is the vector of length n with $(A'B'C')_i = (A'_i, B'_i, C'_i) \in \{0,1\}^3$ for all $i \in 1..n$), the verdict of contributing to neither the numerator nor denominator, just to the denominator, or to both in (†) for the subset of members (A', B', C') of Ξ sharing a common $h_{A'B'C'}$ falls the same easily determined way for every member of the subset. As the cardinalities of these histogram–indexed subsets are also easily computed (see below), one only needs to enumerate the subsets and not individual triplets (A', B', C') of binary vectors. This typically represents enormous savings: with $\vartheta := \{\, h_{A'B'C'} \mid (A', B', C') \in \Xi \,\}$ being the set of all histograms of length n vectors of bit triplets, there holds

$$\#\vartheta = \binom{n + (2^3 - 1)}{2^3 - 1} = \binom{n+7}{7} = \frac{(n+7)!}{7!\, n!},$$

which is asymptotic to $n^7/7!$ as $n \to \infty$ (and is thus polynomial, as opposed to exponential $\#\Xi$) and below 10^{13} when $n = 173$ — still inconveniently large, but much improved over $\sim\!10^{156}$.

Considerable additional savings is possible because one can avoid enumeration (of the typically large fraction) of members of ϑ that contribute to neither the numerator nor denominator of (†). Some additional notation introduced presently by example is helpful: write $n_{A'=1} \in 0..n$ for the number of elements of A' equal to 1, $n_{A'B'=01} \in 0..n$ for the number of elements of $A'B'$ equal to $(0, 1)$, and $n_{A'B'C'=110} \in 0..n$ for the number of elements of $A'B'C'$ equal to $(1, 1, 0)$. Re-express histograms $h_{A'B'C'}$ (previously functions from bit triplets present to $1..n$) as length 8 vectors

$$\begin{aligned}\underline{h}_{A'B'C'} := (&n_{A'B'C'=000}, n_{A'B'C'=001}, n_{A'B'C'=010}, n_{A'B'C'=011},\\ &n_{A'B'C'=100}, n_{A'B'C'=101}, n_{A'B'C'=110}, n_{A'B'C'=111}\ ) \in (0..n)^8\end{aligned}$$

and similarly re-express histograms h_{XY} and h_X for length n binary vectors X and Y as length 4 (in the order $XY = 00,\ 01,\ 10,\ 11$) and length 2 (in the order $X = 0,\ 1$) vectors $\underline{h}_{XY} \in (0..n)^4$ and $\underline{h}_X \in (0..n)^2$, respectively. The set of all histograms of length n vectors of bit triplets is now $\underline{\vartheta} = \{\, \underline{h}_{A'B'C'} \mid (A', B', C') \in \Xi \,\}$ = all length 8 vectors of non-negative integers whose sum of entries is equal to n. Further, given histogram $(n_{000}, n_{001}, n_{010}, n_{011}, n_{100}, n_{101}, n_{110}, n_{111}) := \underline{h} \in \underline{\vartheta}$, the number of length n vectors of bit triplets with it is simply the multinomial coefficient

$$\begin{aligned}N(\underline{h}) &:= \binom{n}{n_{000}, n_{001}, n_{010}, n_{011}, n_{100}, n_{101}, n_{110}, n_{111}}\\ &= n! \,/\, (n_{000}!\, n_{001}!\, n_{010}!\, n_{011}!\, n_{100}!\, n_{101}!\, n_{110}!\, n_{111}!) \geq 1.\end{aligned}$$

If $(n_{000}, n_{001}, n_{010}, n_{011}, n_{100}, n_{101}, n_{110}, n_{111}) = \underline{h}_{A'B'C'}$, then

$$\begin{array}{lllll}
\underline{h}_{A'C'} = (n_{000} + n_{010}, & n_{001} + n_{011}, & n_{100} + n_{110}, & n_{101} + n_{111}), & \\
\underline{h}_{B'C'} = (n_{000} + n_{100}, & n_{001} + n_{101}, & n_{010} + n_{110}, & n_{011} + n_{111}), & \\
\underline{h}_{C'F'} = (n_{000} + n_{010} + n_{100}, & n_{110}, & n_{001} + n_{011} + n_{101}, & n_{111}) & \text{when } F' = f_1\ (A', B'),\\
\underline{h}_{C'F'} = (n_{110}, & n_{000} + n_{010} + n_{100}, & n_{111}, & n_{001} + n_{011} + n_{101}) & \text{when } F' = f_2\ (A', B'),\\
\underline{h}_{C'F'} = (n_{000}, & n_{010} + n_{100} + n_{110}, & n_{001}, & n_{011} + n_{101} + n_{111}) & \text{when } F' = f_3\ (A', B'),\\
\underline{h}_{C'F'} = (n_{010} + n_{100} + n_{110}, & n_{000}, & n_{011} + n_{101} + n_{111}, & n_{001}) & \text{when } F' = f_4\ (A', B'),\\
\underline{h}_{C'F'} = (n_{000} + n_{010} + n_{110}, & n_{100}, & n_{001} + n_{011} + n_{111}, & n_{101}) & \text{when } F' = f_{5'}\,(A', B'),\\
\underline{h}_{C'F'} = (n_{000} + n_{100} + n_{110}, & n_{010}, & n_{001} + n_{101} + n_{111}, & n_{011}) & \text{when } F' = f_{5''}(A', B'),\\
\underline{h}_{C'F'} = (n_{010}, & n_{000} + n_{100} + n_{110}, & n_{011}, & n_{001} + n_{101} + n_{111}) & \text{when } F' = f_{6'}\,(A', B'),\\
\underline{h}_{C'F'} = (n_{100}, & n_{000} + n_{010} + n_{110}, & n_{101}, & n_{001} + n_{011} + n_{111}) & \text{when } F' = f_{6''}(A', B'),\\
\underline{h}_{C'F'} = (n_{000} + n_{110}, & n_{010} + n_{100}, & n_{001} + n_{111}, & n_{011} + n_{101}) & \text{when } F' = f_7\ (A', B'),\\
\underline{h}_{C'F'} = (n_{010} + n_{100}, & n_{000} + n_{110}, & n_{011} + n_{101}, & n_{001} + n_{111}) & \text{when } F' = f_8\ (A', B').
\end{array}$$

Further,

$$
\begin{aligned}
U(C' \mid F') &= \big(H(C') + H(F') - H(C'F')\big)/H(C') \\
&= \big(\nu(n_{C'=0}) + \nu(n_{C'=1}) + \nu(n_{F'=0}) + \nu(n_{F'=1}) - \\
&\qquad \nu(n_{C'F'=00}) - \nu(n_{C'F'=01}) - \nu(n_{C'F'=10}) - \nu(n_{C'F'=11})\big) \bigm/ \big(\nu(n_{C'=0}) + \nu(n_{C'=1})\big) \\
&= \big(\nu(n_{00}+n_{01}) + \nu(n_{10}+n_{11}) + \nu(n_{00}+n_{10}) + \nu(n_{01}+n_{11}) - \\
&\qquad \nu(n_{00}) - \nu(n_{01}) - \nu(n_{10}) - \nu(n_{11})\big) \bigm/ \big(\nu(n_{00}+n_{01}) + \nu(n_{10}+n_{11})\big) \\
&=: U_f(\underline{h}_{A'B'C'})
\end{aligned}
$$

when $\underline{h}_{C'F'} = (n_{00}, n_{01}, n_{10}, n_{11})$ and neither $n_{00} + n_{01}$ nor $n_{10} + n_{11}$ is 0, where $\nu(m) := -(m/n) \cdot \lg(m/n) \geq 0$ for $m \in 1..n$ and $\nu(0) := 0$, and $U(C' \mid F') = 1$ if $n_{00} + n_{01}$ or $n_{10} + n_{11}$ is 0. Hence, as promised earlier, it is a small number of simple operations to calculate $h_{A'C'}$, $h_{B'C'}$, and $U(C' \mid f(A', B'))$ from $h_{A'B'C'}$.

To enumerate all elements $(n_{000}, n_{001}, n_{010}, n_{011}, n_{100}, n_{101}, n_{110}, n_{111})$ of

$$
\underline{\vartheta}_{ABC} := \{\, \underline{h}_{A'B'C'} \mid (A', B', C') \in \Xi \text{ and } h_{A'C'} = h_{AC} \text{ and } h_{B'C'} = h_{BC} \,\} \subseteq \underline{\vartheta}
$$

is simply to ask for all integer solutions to the system

$$
\begin{aligned}
n_{000}, n_{001}, n_{010}, n_{011}, n_{100}, n_{101}, n_{110}, n_{111} &\geq 0 \\
n_{000} + n_{001} + n_{010} + n_{011} + n_{100} + n_{101} + n_{110} + n_{111} &= n \\
n_{000} + n_{010} &= n_{AC=00} \\
n_{001} + n_{011} &= n_{AC=01} \\
n_{100} + n_{110} &= n_{AC=10} \\
n_{101} + n_{111} &= n_{AC=11} \\
n_{000} + n_{100} &= n_{BC=00} \\
n_{001} + n_{101} &= n_{BC=01} \\
n_{010} + n_{110} &= n_{BC=10} \\
n_{011} + n_{111} &= n_{BC=11}
\end{aligned}
$$

of simultaneous mixed linear inequalities and equalities in eight variables (these being the integral points in a particular convex polytope given in $\mathcal{H}$–form), which is easily shown in this case to be the elements of

$$
\begin{aligned}
\{\, &(n_{000},\ n_{AC=01} - n_{BC=11} + n_{111},\ n_{AC=00} - n_{000},\ n_{BC=11} - n_{111}, \\
&\quad n_{BC=00} - n_{000},\ n_{AC=11} - n_{111},\ n_{BC=10} - n_{AC=00} + n_{000},\ n_{111}) \mid \\
&\quad n_{000} \in \max(0, n_{AC=00} - n_{BC=10})..\min(n_{AC=00}, n_{BC=00}) \text{ and} \\
&\quad n_{111} \in \max(0, n_{BC=11} - n_{AC=01})..\min(n_{AC=11}, n_{BC=11}) \qquad \} = \underline{\vartheta}_{ABC}.
\end{aligned}
$$

(Many other expressions are possible.) An easy upper bound for $\#\underline{\vartheta}_{ABC}$ is $(n+1)^2$, which is a comparatively paltry 30,276 when $n = 173$; enumeration is now very computationally feasible:

$$
p_f(A, B, C) = \frac{\text{sum of } \begin{Bmatrix} N(\underline{h}) & \text{if } U_f(\underline{h}) \geq U(C \mid f(A, B)) \\ 0 & \text{otherwise} \end{Bmatrix} \text{ over } \underline{h} \in \underline{\vartheta}_{ABC}}{\text{sum of } N(\underline{h}) \text{ over } \underline{h} \in \underline{\vartheta}_{ABC}}
$$

can be implemented more or less directly (care is needed in numerical details; see below).

The general mathematical strategy presented here also works to compute a number of p–values not used in the main text or described in detail here. (For example, the constraint $h_{A'B'} = h_{AB}$ can be added to the numerator and denominator of (†) or various methods to compensate for large $n_{ABC=000}$ such as constraining $n_{A'B'C'=000} = 0$ or $n_{A'B'C'=001} = 0$ can be tried, etc.) Many of these were experimentally tested, but ultimately $p_f(A, B, C)$ was chosen for the dataset of the main text.

§3. Computational considerations for efficient, accurate calculation of p–values

High–performance manipulation of short vectors of data are provided by the SIMD vector instruction extensions and associated dedicated execution units of modern CPUs (e.g., Apple/IBM/ Freescale AltiVec on PowerPC and MMX/SSE/SSE2/SSE3/SSSE3/SSE4.1/SSE4.2 on Intel x86). In particular, it is profitable to represent the profiles to be analyzed as (generally short) sequences of 128–bit SIMD bitvectors (e.g., two such vectors suffice for $n = 173$). The fast, wide bitwise $\textsc{and}(\cdot,\cdot)$, $\textsc{and}(\cdot,\bar{\cdot})$, $\textsc{or}(\cdot,\cdot)$, $\textsc{xor}(\cdot,\cdot)$, and "population count" (Hamming weight) then available or synthesizable are quite helpful. (The POPCNT instruction of SSE4.2 would be particularly helpful, but unfortunately SSE4.2 is not widely available at this time. However, composing it from other, more widely–available vector instructions is still fast compared to alternatives.)

At times, use of $(n, n_{A'=1}, n_{B'=1}, n_{A'B'=1})$ rather than $\underline{h}_{A'B'}$ for joint histograms of pairs of bitvectors may be convenient. By the combinatorial principle of inclusion–exclusion, conversion in both directions is easy and linear:

$$\begin{bmatrix} n \\ n_{A'=1} \\ n_{B'=1} \\ n_{A'B'=11} \end{bmatrix} = \begin{bmatrix} 1&1&1&1 \\ 0&0&1&1 \\ 0&1&0&1 \\ 0&0&0&1 \end{bmatrix} \begin{bmatrix} n_{A'B'=00} \\ n_{A'B'=01} \\ n_{A'B'=10} \\ n_{A'B'=11} \end{bmatrix} \text{ with inverse}$$

$$\begin{bmatrix} n_{A'B'=00} \\ n_{A'B'=01} \\ n_{A'B'=10} \\ n_{A'B'=11} \end{bmatrix} = \begin{bmatrix} 1&-1&-1&1 \\ 0&0&1&-1 \\ 0&1&0&-1 \\ 0&0&0&1 \end{bmatrix} \begin{bmatrix} n \\ n_{A'=1} \\ n_{B'=1} \\ n_{A'B'=11} \end{bmatrix}.$$

Similarly, $(n,\ n_{A'=1},\ n_{B'=1},\ n_{C'=1},\ n_{A'B'=11},\ n_{A'C'=11},\ n_{B'C'=11},\ n_{A'B'C'=111})$ is an easy and linear reparameterization of $\underline{h}_{A'B'C'}$:

$$\begin{bmatrix} n \\ n_{A'=1} \\ n_{B'=1} \\ n_{C'=1} \\ n_{A'B'=11} \\ n_{A'C'=11} \\ n_{B'C'=11} \\ n_{A'B'C'=111} \end{bmatrix} = \begin{bmatrix} 1&1&1&1&1&1&1&1 \\ 0&0&0&0&1&1&1&1 \\ 0&0&1&1&0&0&1&1 \\ 0&1&0&1&0&1&0&1 \\ 0&0&0&0&0&0&1&1 \\ 0&0&0&0&0&1&0&1 \\ 0&0&0&1&0&0&0&1 \\ 0&0&0&0&0&0&0&1 \end{bmatrix} \begin{bmatrix} n_{A'B'C'=000} \\ n_{A'B'C'=001} \\ n_{A'B'C'=010} \\ n_{A'B'C'=011} \\ n_{A'B'C'=100} \\ n_{A'B'C'=101} \\ n_{A'B'C'=110} \\ n_{A'B'C'=111} \end{bmatrix} \text{ with inverse}$$

$$\begin{bmatrix} n_{A'B'C'=000} \\ n_{A'B'C'=001} \\ n_{A'B'C'=010} \\ n_{A'B'C'=011} \\ n_{A'B'C'=100} \\ n_{A'B'C'=101} \\ n_{A'B'C'=110} \\ n_{A'B'C'=111} \end{bmatrix} = \begin{bmatrix} 1&-1&-1&-1&1&1&1&-1 \\ 0&0&0&1&0&-1&-1&1 \\ 0&0&1&0&-1&0&-1&1 \\ 0&0&0&0&0&0&1&-1 \\ 0&1&0&0&-1&-1&0&1 \\ 0&0&0&0&0&1&0&-1 \\ 0&0&0&0&1&0&0&-1 \\ 0&0&0&0&0&0&0&1 \end{bmatrix} \begin{bmatrix} n \\ n_{A'=1} \\ n_{B'=1} \\ n_{C'=1} \\ n_{A'B'=11} \\ n_{A'C'=11} \\ n_{B'C'=11} \\ n_{A'B'C'=111} \end{bmatrix}.$$

To enable further discussion, a high–level outline of the current GPLv3 C++ implementation for Mac OS X 10.4$^+$ on Intel with GCC from Apple's Developer Tools is below.

- Read and SIMD–ize binary profiles $v_0, \ldots, v_{g-1}$ with $\geq \epsilon$ zeros and $\geq \epsilon$ ones.
- For $a \in 0..(g-1)$, put $A := v_a$:
  - For $b \in (a+1)..(g-1)$, put $B := v_b$:
    - For $c \in 0..(g-1)$ skipping a and b, put $C := v_c$:
      - Note h_{ABC}, $U(C \mid A)$, $U(C \mid B)$, and $U_{\text{obs}}(f) := U(C \mid f(A,B))$ for $f = f_{1/3/5'/5''/7}$.
      - For n_{000} in the appropriate range :
        - For n_{111} in the appropriate range :
          - Note $h_{A'B'C'}$ and $U_{\text{rand}}(f) := U(C' \mid f(A', B'))$ for $f = f_{1/3/5'/5''/7}$.
          - Accumulate multinomial into denominator (common to all logic types).
          - Accumulate multinomial into $f_{1/3/5'/5''/7}$ numerators for those $U_{\text{rand}} \geq U_{\text{obs}}$.
      - Divide numerators by denominator to form $f_{(1\text{–}2)/(3\text{–}4)/(5'\text{–}6'')/(5''\text{–}6')/(7\text{–}8)}$ p–values.
      - For each p–value $\leq \rho$:
        - Resolve f vs. $\overline{f}$ by smaller disagreement with C (keeping both in case of a tie).
        - Note h_{CF} and $U(C \mid F)$, and emit output line if and only if $U(C \mid F) \geq U(C \mid A) + \gamma$, $U(C \mid F) \geq U(C \mid B) + \gamma$, $n_{CF=11} \geq \tau$, $n_{CF=01} < n_{CF=11}$, and $n_{CF=10} < n_{CF=11}$.

(The main text takes $\epsilon := 15$, effectively a lower bound on the entropy of input profiles analyzed.) The final grouping of emitted logic triplets by $\{a, b, c\}$ and selection of a distinguished member from each group is external to the C++ code, easy to implement via sorting, and only takes minutes to process large outputs (e.g., ~2.5 GiB) on a modern PC.

In common use (with $g \gg$ the number of CPUs), parallelization (e.g., for multi–core single computers or for multiple machines) is typically trivial, for example by having different instances complete different intervals of the main $a \in 0..(g-1)$ loop. No cross–instance communication is required (once all instances have completed, simple concatenation of output files suffices). However, work per iteration of the a loop — counting each execution of the c loop as 1 unit of work and ignoring loop overhead — decreases as a increases due to the varying bounds of the b loop. Roughly (relaxing from integers to reals and approximating slightly by taking $a \in [0, g]$ and $b \in [a, g]$), the initial $q \in [0, 1]$ fraction of the a loop represents fraction

$$W(q) := \frac{\int_0^{qg} (g-a)\, da}{\int_0^{g} (g-a)\, da} = \frac{q(2-q)g^2/2}{g^2/2} = q(2-q) \in [0,1]$$

of the work of the whole job, which has inverse $Q(w) = 1 - \sqrt{1-w} \in [0,1]$ for $w \in [0,1]$. Hence, given $m \geq 1$ equally fast CPUs, the a loop should be partitioned so that CPU $i \in 1..m$ computes $a = a_{i-1}..(a_i - 1)$ where integers $0 = a_0 < a_1 < \cdots < a_{m-1} < a_m = g$ are such that $a_i \approx g\, Q(i/m)$. Otherwise (e.g., if approximately equally spaced a_i are used), some CPUs may finish much earlier than others. Of course, if a job queueing system is available and per–slice overhead is negligible, an alternative is to arbitrarily partition (e.g., with approximately equal spacing) the a loop into many more slices than CPUs (perhaps as fine as a single a value per slice, or even further partitioning the b and/or c loops if very large numbers of CPUs are available) and have each CPU work on one slice at a time, assigning slices to free CPUs as slices finish.

For example, on a Mac Pro running OS X 10.4.11 with eight CPUs (2x quad–core 3.0 GHz 64–bit Intel Xeon X5365) and 8x 2 GiB 667 MHz DDR2 ECC FB-DIMMs for the $(n, g) = (173, 2969)$ job of the main text, eight instances of the C++ code as compiled by Apple GCC 4.0.1 (`-m64 -O2`, two SIMD vectors per profile) were run simultaneously with partitioning a = 0..191, 192..397, 398..621, 622..869, 870..1150, 1151..1483, 1484..1918, and 1919..2968. Instances finished in 8 hours 15 minutes ±17 minutes (± ~3.2%). While the current implementation could be further optimized, the running time of the present form is reasonable and has not proven burdensome.

Applied without care, both the finite precision and finite range of commonly–used floating–point arithmetics are troublesome here. For example, 171! (e.g., as numerator in an undesirable method of computing multinomials) exceeds IEEE 754 double's range, and (more unpredictably) representational and accumulated rounding errors may cause incorrect classification of contributions to the numerator of (†) when computing p–values if done directly (which would lead to difficulties in deriving guarantees as to quality of computed p–values). The present implementation addresses these problems for the critical case of deciding contributions to the numerator of (†) with a combination of quick–but–rigorous IEEE 754 extended–precision floating–point interval arithmetic that can fail (and correctly self–diagnose when failure cannot be ruled out) but only rarely in practice, falling back when necessary to relatively–slow–but–exact arbitrary–size integer calculations with GNU MP that cannot fail to resolve numerically difficult cases. The two other key comparisons in the present code — those with ρ and γ — are also guarded with the use of floating–point interval arithmetic, but currently do not have fallback code; numerically unresolved cases are taken to pass but emit with special annotation. As such annotations were not emitted for the run conducted for the main text, the selection of logic triplets for the main text is exactly as specified.

Although IEEE 754 and ubiquitous hardware implementations of it have included support for directed rounding needed for efficient use of interval arithmetic for many years, compiler and runtime environment support generally still lags to this day. For example, aggressively optimizing compilers (including GCC) have a tendency to rewrite floating–point expressions without regard to possible use of non–default rounding modes (or other fine points of IEEE 754), and library functions not infrequently misbehave unpredictably if called with non–default modes in effect. Careful reading of compiler and run–time library documentation (and generally some testing) is needed to determine what compiler switches (e.g., `-frounding-math` is one for Apple GCC 4.0.1) and other strategies (e.g., switching back to standard rounding before calling library functions) seem to be needed to get IEEE 754–compliant behavior; the lack of support for C99 `#pragma`s `STDC FENV_ACCESS` and `STDC FP_CONTRACT` is regrettable. (As a side note regarding compilation options but unrelated to floating–point, higher performance in the current implementation is achieved by allowing more inlining than GCC's defaults permit: increases to `--param max-inline-insns-single`, `--param large-function-growth`, and `--param inline-unit-growth` are helpful.)

Rounding mode changes are generally very expensive on current out–of–order superscalar platforms, making fine–grained switching (necessary to easily abstract with high performance interval arithmetic in high–level languages) undesirable. The current code minimizes mode switching by using the well–known trick of generally leaving rounding set toward $-\infty$ and using extra negations to effect rounding toward $+\infty$. For example, floating–point interval addition $[a, b] + [c, d]$ is performed as $[a \oplus c, -((-b) \oplus (-d))]$, where $\oplus$ is floating–point addition rounding toward $-\infty$. Indeed, one could hope for future hardware implementations that dispense with modes and include all few option bits (especially rounding direction) in the encoding of every instruction; this would reduce the need for programming gymnastics, minimize library and external code issues, be easier on compiler–writers, and simplify microarchitectures.

Accuracy and rounding difficulties with standard library transcendental functions (e.g., the natural logarithms of `std::log`) are avoided by pre–computing tables of $m \cdot \ln(m)$ for $m \in 1..n$ in arbitrary–precision external software (*Mathematica*), rounding values both downward and upward to nearest–representable IEEE 754 extended–precision floating–point numbers to enable interval arithmetic, and placing them into the C++ code as hexadecimal floating–point constants to circumvent problems with accuracy and rounding of decimal floating–point constants (another area in which language work is generally needed). Multinomials are computed by first working in a prime factorization representation of positive integers — storing in packed bitfields exponents of

sufficient primes — with a pre–computed table giving the representation of $m!$ for $m \in 0..n$. As ordinary arithmetic division becomes differences of exponents (and all present divisions will be without remainders), integer SIMD subtractions rapidly form a representation of the desired multinomial coefficient, for which a narrow bounding interval is computed using an additional lookup table of extended–precision hexadecimal floating–point constants and a small number of floating–point multiplications with intermediate values no larger in magnitude than the final multinomial coefficient (so that overflow does not occur as long as the desired coefficient itself is in range).

Finally, many common and/or partially–evaluable sub–expressions (e.g., the p–value denominator shared among all logic types) can be identified, collapsed, and moved outside of as many nested loops as generally possible, either manually or (where possible) by the compiler. □
